# Effect of Gender, Season, and Vitamin D Status on Bone Biochemical Markers in Saudi Diabetes Patients

**DOI:** 10.3390/molecules17078408

**Published:** 2012-07-11

**Authors:** Nasser M. Al-Daghri, Khalid M. Alkharfy, Abdulaziz Al-Othman, Sobhy M. Yakout, Yousef Al-Saleh, Mona A. Fouda, Riad Sulimani, Shaun Sabico

**Affiliations:** 1 Biomarkers Research Program, Department of Biochemistry, College of Science, King Saud University, Riyadh 11451, Saudi Arabia; 2 Prince Mutaib Chair for Biomarkers of Osteoporosis, Biochemistry Department, King Saud University, Riyadh 11451, Saudi Arabia; 3 Department of Clinical Pharmacy, College of Pharmacy, King Saud University, Riyadh 11451, Saudi Arabia; 4 College of Applied Medical Sciences, King Saud University, Riyadh 11451, Saudi Arabia; 5 College of Medicine, King Saud University for Health Sciences, Riyadh 11426, Saudi Arabia; 6 Medicine Department, King Khalid University Hospital, Riyadh 11411, Saudi Arabia

**Keywords:** vitamin D status, osteocalcin, osteoprotegerin, season, Saudi Arabia

## Abstract

Biochemical bone turnover markers (BTMs) provide important information on the diagnosis, therapy and monitoring of metabolic bone diseases. They are evident before measurable changes in bone mineral density (BMD) take place. A total of 35 adult Saudi patients (23 males; 12 females) with type 2 diabetes and diagnosed to be vitamin D deficient were recruited in this prospective study. Here we investigated the effects of gender, season, and vitamin D status on bone biochemical markers of bone remodeling. Anthropometry and blood samples were collected at different intervals. Metabolic parameters and bone biomarkers were measured routinely and by ELISA. Both males and females had a significant increase in their vitamin D status over time, but no significant changes in the bone biomarkers were observed in females. In males there was a significant increase in circulating levels of corrected calcium and OPN (*p* = 0.004 and 0.01 respectively) and a significant decrease in crosslaps (*p* = 0.005). In all subjects there was a modest but significant positive relationship between vitamin D status and OC (R = 0.34; *p* = 0.04). In conclusion, our study demonstrates that changes in bone remodeling markers are affected by season, gender, and possibly vitamin D status. This gender difference may well reflect the physiologic pathway responsible for the higher peak bone mass achieved in males compared to females.

## 1. Introduction

In recent years, biochemical markers of bone metabolism have been shown to provide substantial information in studies of pathophysiology and assessment of various metabolic bone diseases, including osteoporosis [1]. The bone markers have potential clinical value in risk evaluation and monitoring of treatment in osteoporosis, rheumatic diseases, metabolic bone diseases and skeletal metastases [2].

The effects of physical activity on bone density can be evaluated by direct measurement of bone mineral density (BMD) by means of radiological methods at different sites [3,4]. Acute responses can be assessed by measuring the serum level of biochemical markers of bone turnover, which reﬂect cellular events involved in bone metabolism [5]. Since biochemical alterations are evident before measurable changes in BMD take place, alterations in isolated markers of bone turnover are useful for evaluating the impact of a speciﬁc intervention on bone metabolism [6]. Routine clinical use is, however still controversial [7,8], partly due to lack of information on the variability of bone marker levels. The new biochemical markers developed recently have markedly improved the sensitivity and specificity for reflecting the overall rate of bone formation or resorption. Formation markers are products of active osteoblast expressed during different phases of osteoblast development and measured in terms of concentration (*i.e.*, osteocalcin, OC). The resorption markers are degradation products of bone collagen that can be quantiﬁed from serum or urine (*i.e.*, C-terminal cross-linked telopeptides of type I collagen—CTx) [5,9].

Bone remodeling processes are under the influence of several hormones, the action of which might be modulated by osteoprotegerin (OPG) and osteopontin (OPN) which are considered as bone metabolism biomarkers. OPG is the main osteoclastogenesis modulator. It is also a member of tumor necrosis factor receptor (TNFR) super family produced by a variety of tissues including the cardiovascular system (heart, arteries, veins), lungs, kidneys, bones as well as hematopoietic and immune cells [10]. OPG acts as a soluble decoy receptor for receptor activator of nuclear factor κ-B ligand (RANKL) and neutralizes this essential cytokine required for osteoclast differentiation [11]. Increased OPG levels have been observed in men with advanced coronary artery disease (CAD) [12]. OPN on the other hand is secreted as a calcium-binding glycophosphoprotein that has been implicated in bone remodeling and inflammation. Similar to OPG, osteopontin is widely distributed in different human cells including osteoblasts, lymphocytes, macrophages, endothelial cells and vascular smooth muscle cells [13].

Studies showed that vitamin D status plays a key role for general health and particularly for the bone even if it is highly variable, and depends on outdoor sunlight exposure time (during peak sunlight) and latitude [14].

Most studies have examined seasonal changes in serum 25OHD and serum PTH, but there are limited studies that examined corresponding changes in biochemical markers of bone turnover and made contrasting observations [15,16]. Woitge *et al.* [16] demonstrated in healthy subjects that specific markers of bone turnover show significant seasonal changes that are directly related to variations in the hormonal regulation of skeletal homeostasis. Seasonal variation in bone mass has also been reported, with bone mass decreasing in winter and increasing in summer periods [15,17]. This observation however has not been replicated [18,19]. We hypothesized that in elderly women, seasonal changes in serum 25OHD are associated with parallel changes in serum PTH. The change in serum PTH contributes to bone resorption, indicated by changes in biochemical markers of bone turnover leading to decreased BMD. Our study systematically investigated the effect of gender, season, and vitamin D status on bone biochemical markers of bone remodeling. We have attempted to define the relationships among vitamin D, season and gender to clarify the influence of these factors on markers of bone formation and resorption over time.

## 2. Results

**Figure 1:**
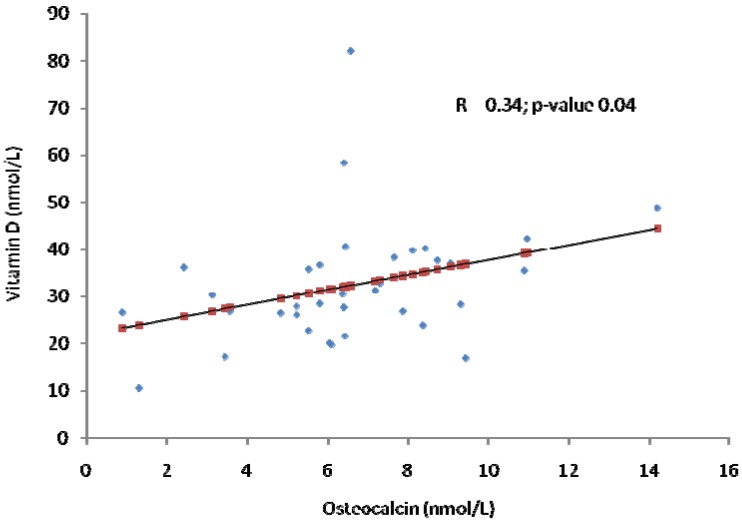
Modest association between vitamin D and osteocalcin (all subjects).

Table 1 describes the baseline anthropometric and biochemical parameters of male and female subjects included in the study. Females had significantly higher levels of vitamin D and OPN as compared to males (both *p* < 0.001, respectively). On the other hand, males had significantly higher OC than females (*p* < 0.001). Table 2 shows the seasonal comparisons of the parameters measured across all subjects. Most of the parameters were comparable, with the exception of corrected calcium which showed a significantly increasing trend (*p* = 0.012) as well as OPN (*p* = 0.001). Vitamin D status was significantly higher during summer as compared to baseline winter, which was probably due to the advice to increase sun exposure (*p* < 0.001). Table 3 shows the same comparisons, but stratified to gender. While both males and females had a significant increase in their vitamin D status over time, no significant changes in the bone biomarkers were observed in females. In males however there was a significant increase in circulating levels of corrected calcium and OPN (*p* = 0.004 and 0.01 respectively) and a significant decrease in crosslaps (*p* = 0.005). Figure 1 shows a modest but significant positive relationship between vitamin D status and OC (R = 0.34; *p* = 0.04). 

**Table 1:** Baseline Anthropometric and Biochemical Characteristics of Male and Female Patients.

	Winter_1 Males	Winter_1 Females
N	23	12
Age (years)	57.1 ± 7.7	43.9 ± 13.2
BMI (kg/m^2^)	29.7 ± 1.4	33.4 ± 1.2
Systolic BP (mmHg)	116.2 ± 3.2	128.8 ± 5.3
Diastolic BP (mmHg)	78.1 ± 1.8	80.0 ± 2.8
Glucose (mmol/L)	10.0 ± 0.24	9.4 ± 0.13
Triglycerides (mmol/L)	1.8 ± 0.19	2.7 ± 0.82
Total Cholesterol (mmol/L)	5.1 ± 0.35	5.6 ± 0.37
Calcium (mmol/L)	2.4 ± 0.11	2.5 ± 0.02
Corrected Calcium (mmol/L)	2.3 ± 0.06	2.5 ± 0.03
25-OH Vitamin D (nmol/L)	**29.8 ± 0.10**	35.6 ± 0.10 *
Osteocalcin (ng/mL)	**6.5 ± 0.10**	4.4 ± 0.24 *
Osteoprotegerin (pg/mL)	533.5 (274)	559.9 (392)
Osteopontin (ng/mL)	**1.8 ± 0.25**	3.4 ± 0.14 *
Crosslaps (pg/mL)	0.27 ± 0.03	0.23 ± 0.04

Notes: Data presented as mean ± SD; * denotes significance at *p* < 0.05.

**Table 2:** Effect of Season and Vitamin D on Bone Markers.

	Winter_1	Summer	Winter_2	*p*-value
N	35	35	35	
Gender (M/F)	23/12			
Age (years)	52.6 ± 11.6			
BMI (kg/m^2^)	31.6 ± 3.8	31.4 ± 5.2	31.3 ± 5.4	0.84
Systolic BP (mmHg)	123.0 ± 14.5	125.8 ± 8.7	122.9 ± 11.0	0.60
Diastolic BP (mmHg)	79.1 ± 7.1	79.4 ± 7.4	76.5 ± 7.0	0.33
Glucose (mmol/L)	9.8 ± 2.0	12.2 ± 1.6	11.1 ± 1.6	0.29
HDL-Cholesterol (mmol/L)	0.71 ± 0.33	1.0 ± 0.15	0.91 ± 0.10	0.87
Insulin (IU/mL)	22.6 ± 1.3	31.0 ± 1.4	33.2 ± 1.6	0.06
Calcium (mmol/L)	2.4 ± 0.32	2.4 ± 0.11	2.5 ± 0.25	0.11
Corrected Calcium (mmol/L)	2.3 ± 0.22	2.4 ± 0.13 *	2.5 ± 0.19 ^#^	**0.012**
25-OH Vitamin D (nmol/L)	31.0 ± 1.3	64.0 ± 1.4 *	53.7 ± 1.2 *	**<0.001**
Osteocalcin (ng/mL)	5.7 ± 1.6	7.0 ± 1.7	5.7 ± 1.8	0.27
Osteoprotegerin (pg/mL)	527.1 ± 1.4	489.2 ± 1.4	476.3 ± 1.4	0.31
Osteopontin (ng/mL)	2.3 ± 0.46	4.2 ± 0.36	5.5 ± 0.56 *	**0.011**
Crosslaps (ng/mL)	0.25 ± 0.03	0.15 ± 0.04	0.28 ± 0.1	0.28

Data represented as Mean ± SD; * Group is significantly different from First Winter; ^#^ Group is significantly different from Summer.

**Table 3:** Effect of Season on Bone Markers in Males and Females.

	Males				Females			
	Winter_1	Summer	Winter_2	*p*-value	Winter_1	Summer	Winter_2	*p*-value
N	23				12			
Age (years)	57.1 ± 7.7				43.9 ± 13.2			
BMI (kg/m^2^)	29.7 ± 1.4	28.5 ± 1.9	28.5 ± 2.0	0.70	33.4 ± 1.2	34.3 ± 1.4	34.1 ± 1.4	0.47
Systolic BP (mmHg)	116.2 ± 3.2	120.0 ± 2.6	120.0 ± 4.2	0.57	128.8 ± 5.3	131.1 ± 2.0	125.5 ± 3.4	0.58
Diastolic BP (mmHg)	78.1 ± 1.8	76.2 ± 2.6	77.5 ± 2.5	0.80	80.0 ± 2.8	82.2 ± 2.2	75.5±2.4	0.27
Glucose (mmol/L)	10.0 ± 0.24	12.9 ± 0.14	11.2 ± 0.15	0.49	9.4 ± 0.13	11.0 ± 0.12	11.0 ± 0.11	0.45
Triglycerides (mmol/L)	1.8 ± 0.19	2.2 ± 0.25	2.3 ± 0.46	0.28	2.7 ± 0.82	2.1 ± 0.21	1.9 ± 0.17	0.54
Total Cholesterol (mmol/L)	5.1 ± 0.35	5.1 ± 0.47	4.8 ± 0.21	0.86	5.6 ± 0.37	5.1 ± 0.29	5.0 ± 0.32	0.16
Calcium (mmol/L)	2.4 ± 0.11	2.4 ± 0.03	2.6 ± 0.07	0.08	2.5 ± 0.02	2.4 ± 0.05	2.4 ± 0.07	0.24
Corrected Calcium (mmol/L)	2.3 ± 0.06	2.4 ± 0.03	2.5 ± 0.05 *^#^	**0.004**	2.5 ± 0.03	2.3 ± 0.05	2.5±0.06	0.07
Vitamin D (nmol/L)	29.8 ± 0.10	61.7 ± 0.11 *	52.6 ± 0.09 *	**<0.001**	35.6 ± 0.10	72.3 ± 0.16	57.9 ± 0.09 *	**0.02**
Osteocalcin (ng/mL)	6.5 ± 0.10	9.0 ± 0.11	7.3 ± 0.12	0.11	4.4 ± 0.24	2.3 ± 0.20	1.9 ± 0.25	0.87
Osteoprotegerin (pg/mL)	533.5 (274)	558.3 (294)	487.9 (278)	0.45	559.9 (392)	420.2 (309)	436.1 (248)	0.29
Osteopontin (ng/mL)	1.8 ± 0.25	4.5±0.21	6.2 ± 0.29 *	**0.01**	3.4 ± 0.14	3.7 ± 0.26	4.3 ± 0.15	0.59
Crosslaps (ng/mL)	0.27 ± 0.03	0.16 ± 0.03 *	0.18 ± 0.02	**0.005**	0.23 ± 0.04	0.15 ± 0.03	0.17 ± 0.04	0.12

Repeated Measures Analysis of Variance.

## 3. Discussion

The main findings of the present study is that while female subjects with diabetes have significantly higher vitamin D levels as compared to males in all different time points, changes in bone biomarkers were more apparent in males, suggesting not only gender difference in the expression of bone biomarkers independent of season, but also indicating that females might need a much higher level of vitamin D to elicit the desired effects in bone biomarker changes.

Similar findings have been reported from New Zealand and Brazil, where middle aged and older men had significantly lower levels of 25(OH)D than women [20,21]. Furthermore in all subjects there was a slight decrease in the median serum vitamin D concentration during the 2nd winter when compared to summer but still greatly higher the baseline. This is an expected result, which suggests that the subject under study has increased levels of vitamin D acquired through sunlight exposure and diet compared with baseline winter. This poses the question as to whether the Saudi population has any significant exposure to sunlight; otherwise a high rise in median serum vitamin D levels would be expected during the summer period. 

The seasonal variation in 25-OHD levels and the trend towards an association between serum 25-OHD levels and time spent outdoors suggest that production of vitamin D in the skin is the principal determinant of 25-OHD levels. These findings are consistent with research findings regarding the overall Australian population [22] and African Americans [23]. It is likely that time spent outdoors, particularly if it includes weight-bearing exercise, will have health benefits in addition to those associated with increased vitamin D production.

OC, however, seems to diverge from the pattern, with peak values in summer (Table 2). Discordance in seasonal changes between the bone formation markers has been reported before [2,24]. The reason for this difference is unclear and warrants further investigation to clarify the responsible mechanism(s). Some consistent patterns nevertheless did emerge. For example, when analyzed osteocalcin values were greater in men than in women. Our findings of higher bone formation marker values in men than women around the age of 50 years confirm smaller studies that have compared these markers between genders [25,26]. Likewise, majority of previous studies observed an increase in both OC and bone ALP around the time of menopause, at least in white Caucasian women [27,28]. Higher bone formation markers have also been noted in adult males as compared to female subjects [29]. The gender effect on bone formation marker may also be explained by differences in sex steroids, as shown by studies revealing that the best predictors of bone markers were testosterone levels in boys [30,31] and estrogen in girls [32]. 

It is well known that vitamin D deficiency causes an increase in bone resorption (presumably from the rise in PTH caused by hypocalcaemia). Subgroup analysis revealed that the time profile of vitamin D is inversely correlated with that of CTX in both males and females. There is a significant difference in men in summer as compared to baseline winter. In contrast, our data did not show a significant change in CTX during all time period for both genders. These differences in the profiles of the various biochemical markers of bone resorption (CTX) in male and female may reflect their relative specificity for collagen *versus* bone. The CtX level in both summer and 2nd winter are lower than the base line winter in both gender. The increased consumption of both Ca and proteins may well have played a major role in the inhibition of PTH and consecutive lowering of the CTX markers of bone resorption. Furthermore, others have previously shown that markers of bone resorption are subject to significant diurnal variation [33,34]. Bone resorption is thus at its maximum early in the morning (05:00–08:00) and declines to a minimum in the late afternoon.

Within the bone marrow, the most prominent source of OPN is osteoblasts, which produce OPN while forming bone. The resulting OPN comprises approximately 2% of the non-collagenous protein in bone [35]. In addition to osteoblasts, pre-osteoblasts, osteocytes as well as other haemopoietic cells, also synthesise OPN within the BM [36]. The expression of OPN and other non-collagenous proteins, such as osteocalcin, is highly regulated throughout osteoblast differentiation. OPN, induced in response to 1,25(OH)2D3 in osteoblasts, has been reported to modulate bone mineralization.

Regardless, gender effect OPG decreases with increase of vitamin D as shown in Table 1. OPG is a cytokine that belongs to the tumour necrosis factor receptor family and a decoy receptor that blocks the interaction of RANK with its ligand (RANKL), thus inhibiting osteoclast differentiation and activity.

In regression analyses, gender was the consistent correlate of all markers of bone remodeling. The gender difference is an interesting finding that was clearly investigated and documented in previous studies [37]. This gender difference seemed to be independent of body size as assessed by insignificant variation for BMI in our study. It may reflect gender differences in calcium intake and vitamin D levels as we showed, higher muscle mass and lower fat mass in males, differences in growth rates, cultural lifestyle (specifically in this part of the world), or more interestingly the genetic difference, explaining peak bone mass variation [38].

The present study has some limitations. Findings are at best suggestive since the study population may not be representative of the general adult population in Saudi Arabia due to sample size and lack of non-diabetic group. However, this study was more comprehensive than earlier research and collected data on body composition components, muscle strength, physical activity, PTH, and biomarkers of bone formation and resorption in relation to vitamin D status that were not previously evaluated.

## 4. Methodology

### 4.1. Subjects

A total of 35 (23 male; 12 female) adult Saudi patients with type 2 diabetes aged 40 years and above, and diagnosed to be vitamin D deficient (defined as having less than 20 ng/mL of 25 hydroxyvitamin D [25(OH)D]) were recruited for the study. Written and informed consents were obtained before inclusion. Ethics approval was granted by the Ethics Committee of the College of Science, King Saud University, Riyadh, Kingdom of Saudi Arabia (KSA). Participating subjects were recruited and enrolled longitudinally in 4 primary health care centers (PHCCs) within the Riyadh Central Region during the summer months (April–July 2009). They were asked to complete a generalized questionnaire, which contains demographic information including past and present medical history, and to return after overnight fasting for more than 10 h for anthropometry and blood withdrawal. They were also seen 6 and 12 months later for another anthropometry and metabolic assessments.

### 4.2. Anthropometry and Blood Collection

Subjects were requested to visit their respective PHCCs in an overnight fasted state (>10 h) for anthropometry and blood withdrawal by the PHCC nurse and physician on duty, respectively. Anthropometry included height (rounded off to the nearest 0.5 cm), weight (rounded off to the nearest 0.1 kg), waist and hip circumference (centimeters), and mean systolic and diastolic blood pressure (millimeters of Hg) (average of 2 readings). Body mass index was calculated as weight in kilograms divided by height in square meters. Overweight was defined as having a BMI of 25 to 29.9 kg/m^2^; obesity, at least 30 up to 34.9; and morbid obesity, at least 35. Fasting blood samples were collected and transferred immediately to a non-heparinized tube for centrifugation. Collected serum was then transferred to pre-labeled plain tubes; stored in ice; and delivered to the Biomarkers Research Program (BRP) in King Saud University, Riyadh, KSA, for immediate storage at −20 °C.

### 4.3. Sunlight Exposure and Vitamin D Diet

Subjects were given verbal advice to expose themselves to sunlight for 5 to 30 min twice a week either before 10:00 AM and/or after 3:00 PM. The time for sun exposure was based on a previously reported study [39] detailing the hours of daylight in Riyadh, KSA, during which ultraviolet radiation levels are considered carcinogenic and thus should be avoided. They were also regularly encouraged every week through Short Message Service (SMS) to include in their diet increased amounts of vitamin D-rich foods, such as cod liver oil, salmon, tuna, cow liver, dairy products, and vitamin D-fortified foods. To ensure compliance, they were instructed to keep a diary in which they recorded sun exposure times and outdoor physical activity; such diaries were submitted at the end of the study period.

### 4.4. Sample Analyses

Fasting glucose, lipid profile, calcium, and phosphorous were measured using a chemical analyzer (Konelab, Espoo, Finland). Serum 25(OH)D was measured by a specific enzyme-linked immunosorbent assay (IDS, Tyne and Wear, UK). The inter- and intra-assay variability of this assay was 5.3% and 4.6%, respectively. Caution was exercised in the interpretation of results, as significant variability between different assays and laboratories has been reported [15]. It is noted that although the BRP laboratory did not participate in the Vitamin D External Quality Assessment Scheme, Quality Assurance (QA) standards are maintained by ISO 9000 and 17025, whereas the QA department audits the BRP laboratory at regular intervals.

C-Terminal cross-linked telopeptide of type I collagen (CrossLaps, also known as b-CTx) assays were performed with a serum crossLaps_ ELISA kit (Immunodiagnoztic AG, Bensheim, Germany). Maximum intra- and inter-assay CVs were 3.0 and 10.9%, respectively, and the detection limit was 0.020 ng/mL.

Other bone markers (OC, OPG, and OPN) were measured by Luminex IS 200 (Luminexcorp, Austin, TX, USA) the protocol was performed as per the manufacturer’s instruction (Millipore, Billerica, MA, USA) with detection across a range of 1–10,000 pg/mL for each analyte.

### 4.5. Data Analysis

Data were analyzed using the Statistical Package for the Social Sciences version 16.0 (SPSS, Chicago, IL, USA). Normal continuous variables were presented as mean ± standard deviation. Independent T-test was done to compare gender differences during baseline winter. Repeated measures analysis of variance (ANOVA) was done to compare differences between groups. Regression analyses were performed on selected dependent variables of interest (HDL-cholesterol and corrected calcium) using serum 25(OH)-vitamin D as an independent variable. Significance was set at *p* < 0.05.

## 5. Conclusion

In conclusion, our study demonstrates changes in bone remodeling markers that are affected by season, gender, and possibly vitamin D status. Gender is a consistent independent correlate of all markers of remodeling measured in this study. This gender difference in remodeling markers during season may well reflect the physiologic pathway responsible for the higher peak bone mass achieved in males compared to females. That, however, remains to be proven in longitudinal studies.
